# Knockdown of the salivary protein gene NlG14 caused displacement of the lateral oviduct secreted components and inhibited ovulation in *Nilaparvata lugens*

**DOI:** 10.1371/journal.pgen.1010704

**Published:** 2023-04-03

**Authors:** Haoli Gao, Huihui Zhang, Xiaowei Yuan, Xumin Lin, Jianzheng Zou, Na Yu, Zewen Liu

**Affiliations:** Key laboratory of Integrated Management of Crop Diseases and Pests (Ministry of Education), College of Plant Protection, Nanjing Agricultural University, Weigang 1, Nanjing, China; University of Kentucky, UNITED STATES

## Abstract

Saliva plays important roles in insect feeding, but its roles in insect reproduction were rarely reported. Here we reported that the knockdown of a salivary gland-specific gene *NlG14* disrupted the reproduction through inhibiting the ovulation of the brown planthopper (BPH), *Nilaparvata lugens* (Stål), one of the most devastating rice pests in Asia. *NlG14* knockdown caused the displacement of the lateral oviduct secreted components (LOSC), leading to the ovulation disorder and the accumulation of mature eggs in the ovary. The RNAi-treated females laid much less eggs than their control counterparts, though they had the similar oviposition behavior on rice stems as controls. NlG14 protein was not secreted into the hemolymph, indicating an indirect effect of *NlG14* knockdown on BPH reproduction. *NlG14* knockdown caused the malformation of A-follicle of the principal gland and affected the underlying endocrine mechanism of salivary glands. NlG14 reduction might promote the secretion of insulin-like peptides NlILP1 and NlILP3 from the brain, which up-regulated the expression of Nllaminin gene and then caused the abnormal contraction of lateral oviduct muscle. Another explanation was NlG14 reduction disrupted the ecdysone biosynthesis and action through the insulin-PI3K-Akt signaling in ovary. Altogether, this study indicated that the salivary gland specific protein NlG14 indirectly mediated BPH ovulation process, which established a connexon in function between insect salivary gland and ovary.

## Author summary

Salivary glands play important roles in the feeding process of animals. The salivary glands of insects can secrete various proteins through the exocrine pathway to help them digest and regulate the defense response of host. The endocrine function of insect salivary glands is rarely reported. We found that decreased expression of the salivary protein gene NlG14 resulted in abnormal contraction of lateral oviduct muscles, thus inhibiting ovulation of the brown planthopper. NlG14 could not be secreted into the haemolymph as an endocrine function, but NlG14 knockdown caused the deformity of salivary gland. The reduction of NlG14 regulated insulin release in the brain and insulin-PI3K-Akt signaling in ovary. The disruption of these signaling pathways caused the abnormal contraction of lateral oviduct muscles, resulting in ovulation disorders. These findings provide a case of salivary gland regulating ovarian reproduction in insects.

## Introduction

The functions of insect salivary glands have been widely reported, such as secreting various digestive enzymes to help digest food [1], secreting glue protein used to affix a newly formed puparium to a substrate [2], and secreting gelling saliva to help fix and lubricate the stylet [3]. The secreted salivary proteins or RNAs were involved in their interaction with host plants [4–6]. These functions depend on the exocrine mechanism of the salivary gland to transport products through the ducts to the lumen of the salivary gland. The endocrine function of insect salivary glands is poorly understood and has been only demonstrated in *Drosophila* recently: salivary gland-derived secreted factor (Sgsf) peptide was secreted into hemolymph, which regulated Dilp2 secretion in the brain [7].

The brown planthopper (BPH), *Nilaparvata lugens* (Stål) (Hemiptera: Delphacidae), is one of the most important insect pests on rice [8]. BPH uses stylet to suck sap from rice stem and secrets saliva to host plants to regulate plant defense responses [3,9]. In the process, it can also transmit rice viruses and cause serious yield and economic losses [10]. The clear 3D reconstruction model showed that BPH salivary glands were composed of the principle gland, accessory gland, and duct, among which the principle gland consisted nine types of follicles (follicle A-I) [11]. Follicles H was previously known as A-follicle of the principal gland (APG) [12]. We have previously shown that APG is the main localization site of NlG14 protein, an important salivary protein involved in the interaction between BPH and rice, but its physiological function in BPH remains unclear [13].

Reproduction is a fundamental feature and the most important stage for life cycle of insects. Ovulation is a process of expulsion of oocytes from the ovary into the oviducts, which is an essential step in the reproductive process. In insects, ovulation is regulated by several signaling molecules, such as 20-hydroxyecdysone (20E), juvenile hormones (JH), octopamine, and ovulin [14–17]. In addition, ovarian muscle contraction involving laminin and integrin plays an important role in ovulation and egg shape maintenance. RNA interference (RNAi) of NlOsdp, a lateral oviduct secreted protein of BPH ovary, inhibited oocyte delivery from the lateral oviduct to the common oviduct [18]. Although there were many reports on the function of salivary glands in BPH [3,19,20], whether they have effects on ovulation is not known.

In this study, we found that knockdown of the salivary gland-specific NlG14 gene caused the abnormal secretion of proteins in the lateral oviduct and ovulation disorder in female adults, ultimately leading to the accumulation of mature eggs in ovary in BPH. NlG14 protein was not secreted into hemolymph to directly affect ovary functions, but instead might produce the effects through insulin-PI3K-Akt signaling pathway through up-regulating NlILP1 and NlILP3 in the brain. These findings provide a case of salivary gland regulating ovarian reproduction in insects.

## Results

### Knockdown of NlG14 caused displacement of the lateral oviduct secreted components (LOSC) in *Nilaparvata lugens*

**Fig 1:**
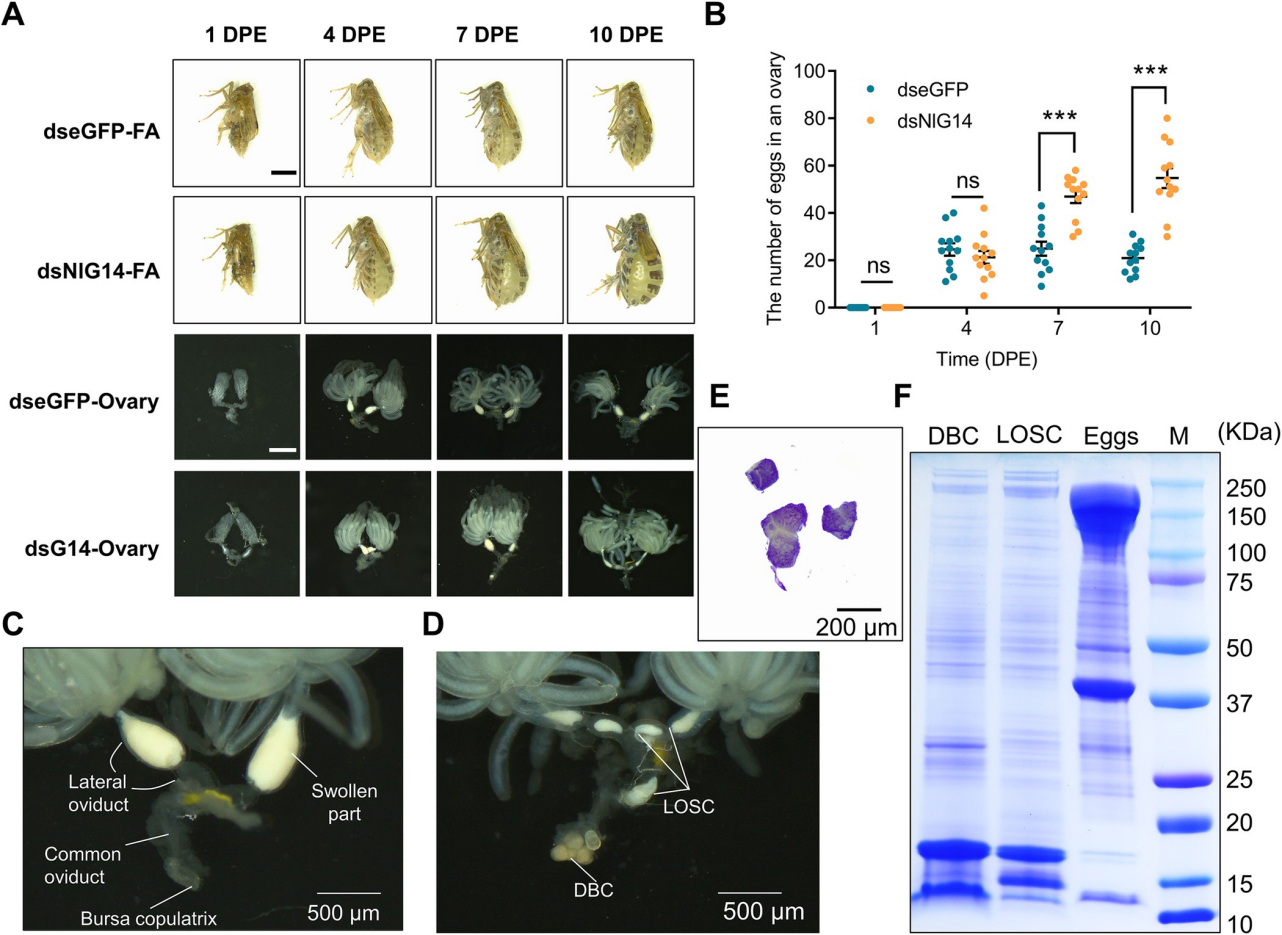
Knockdown of NlG14 caused ovulation deficiency and displacement of Lateral oviduct secreted components (LOSC). (A) Representative images of ovaries and females treated by dsNlG14 or control dseGFP on the 1, 4, 7, and 10 day post-eclosion (DPE). Scale bars are 1 mm. (B) Number of eggs in ovary of virgin females injected with dsNlG14 or dseGFP on 1, 4, 7, and 10 DPE. Data are mean±SE (n = 20). (C, D) Oviduct phenotypes from dseGFP-treated (C) and dsNlG4-treated (D) females on 7 DPE. (E) Coomassie staining of DBC. (F) SDS-PAGE analysis of DBC, LOSC and eggs. DBC, deposits in bursa copulatrix; LOSC, lateral oviduct secreted components. Significant differences were determined using Student’ s t-test: ***P<0.001; ns, no significance.

Our previous studies demonstrated that the salivary protein NlG14 from BPH induced defense responses in plant and the knockdown of NlG14 significantly affected the survival of BPH nymphs [13]. To test whether NlG14 affected the ovary development by affecting food intake, we performed developmental analysis of the ovary. Egg numbers remained in the ovaries of BPH injected with dsNlG14 increased gradually within 10 days, and the abdomen of those BPH gradually inflated due to the accumulation of mature eggs (Fig 1A and 1B).

Each lateral oviduct of BPH can be divided into two parts, a semitransparent base attached to the common oviduct and a swollen part filled with opaque gel-like material near the ovary. The swollen part of the lateral oviducts is a unique structure in BPH and contains various lateral oviduct secretory proteins which are essential for normal ovulation [18]. Here we found that the lateral oviduct secreted components (LOSC) in the swollen part of ovaries in BPHs injected with dsNlG14 were significantly reduced and transferred to the semitransparent base of the lateral oviduct (Fig 1C and 1D).

The bursa copulatrix is an organ in which females store male semen, staying semitransparent when not fertilized (Fig 1C). We observed the deposition of solid granular material in the bursa copulatrix of dsNlG14-treated BPH females (Fig 1D). We speculated that the deposit in bursa copulatrix (DBC) was made of proteins, possibly from the LOSC or the eggs. To test our hypothesis, the Coomassie staining was performed on DBC. The result showed that DBC was significantly stained, indicating that it was made of proteins (Fig 1E). SDS-page results showed that the protein bands in DBC and LOSC were more similar than that between DBC and eggs (Fig 1F). The LC-MS results of DBC and LOSC showed that the top two abundant proteins (shematrin-like protein and vitellogenin-like) were consistent. Furthermore, 14 of the top 20 abundant protein were the same, and 62 of the top 100 abundant protein were the same. (S2 Table). The different components between DBC and LOSC might be from their existences in different ovary parts with different physiological conditions, in which the surrounding proteins might be adhered. These results indicated that DBC were formed due to the abnormal deposition of LOSC.

### NlG14 knockdown affected the contraction of the muscles of lateral oviduct

**Fig 2:**
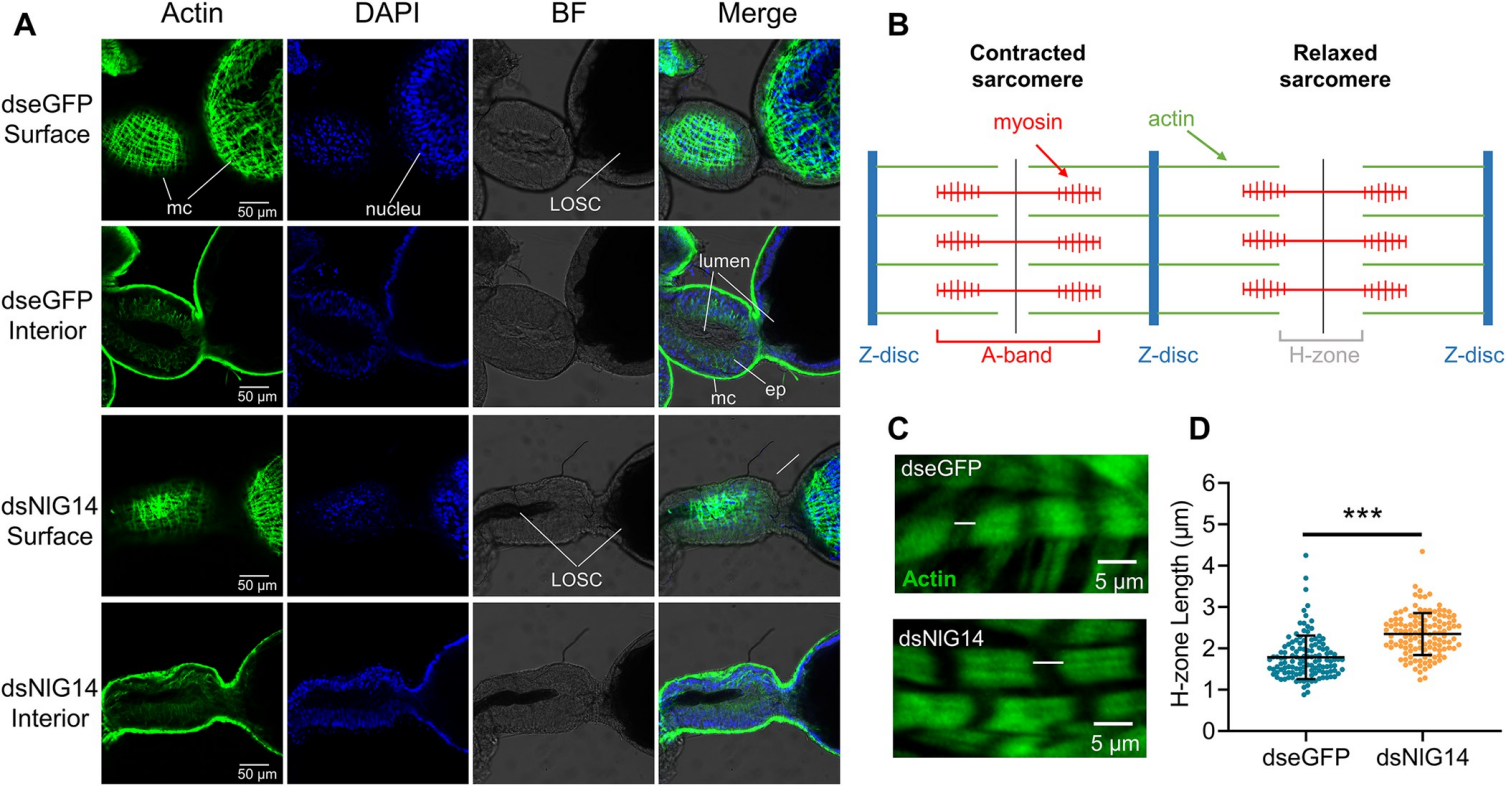
Knockdown of NlG14 effected lateral oviduct contractions. (A) Representative images of lateral oviduct treated by dsNlG14 or control dseGFP on the 7 day post-eclosion (DPE). The surface of the lateral oviduct is covered with a muscle cell (mc) network. Internal focus showed that the lateral oviduct is composed of the epithelium (ep) and lumen. The lateral oviduct secreted components (LOSC) in the swollen part of ovaries in BPHs injected with dsNlG14 were transferred to the semitransparent base of the lateral oviduct. (B) The sliding filament model for muscle contraction. (C) Representative images of sarcomere structure in the lateral oviduct muscle. Actin (green) are marked with a FITC-Phalloidin. (D) H zone length is increased under knockdown of NlG14. Data are mean ± SD (n = 125 sarcomeres). Significant differences were determined using Student’ s t-test: ***P<0.001.

To further investigate the reason of LOSC displacement, the immunofluorescence staining was performed on the lateral oviduct. Confocal laser scanning observation showed that the surface of the lateral oviduct was covered with a muscle cell network, and the interior was mainly composed of the lumen and epithelium (Fig 2A). The lateral oviduct secreted components (LOSC) in the swollen part of ovaries in BPHs injected with dsNlG14 were transferred to the semitransparent base of the lateral oviduct (Fig 2A). The sarcomere is the basic contractile unit in muscle (Fig 2B). It is composed of thick filaments of myosin (the A-band) and thin filaments of actin. The thin filaments are attached to Z-discs at the sarcomere boundaries. During the contraction of muscle cells, myosin pulls on the actin, decreasing the length of H-zone. Thus, we examined the length of the H-zone of the muscle of lateral oviduct in dsNlG14 treatment and the control on the 7 DPE (Fig 2C). The average length of H-zone increased when compared with the control (Fig 2D). This organization suggested that NlG14 knockdown affected the contraction of the muscles of lateral oviduct.

### NlG14 knockdown caused ovulation deficiency in *N*. *lugens*

**Fig 3:**
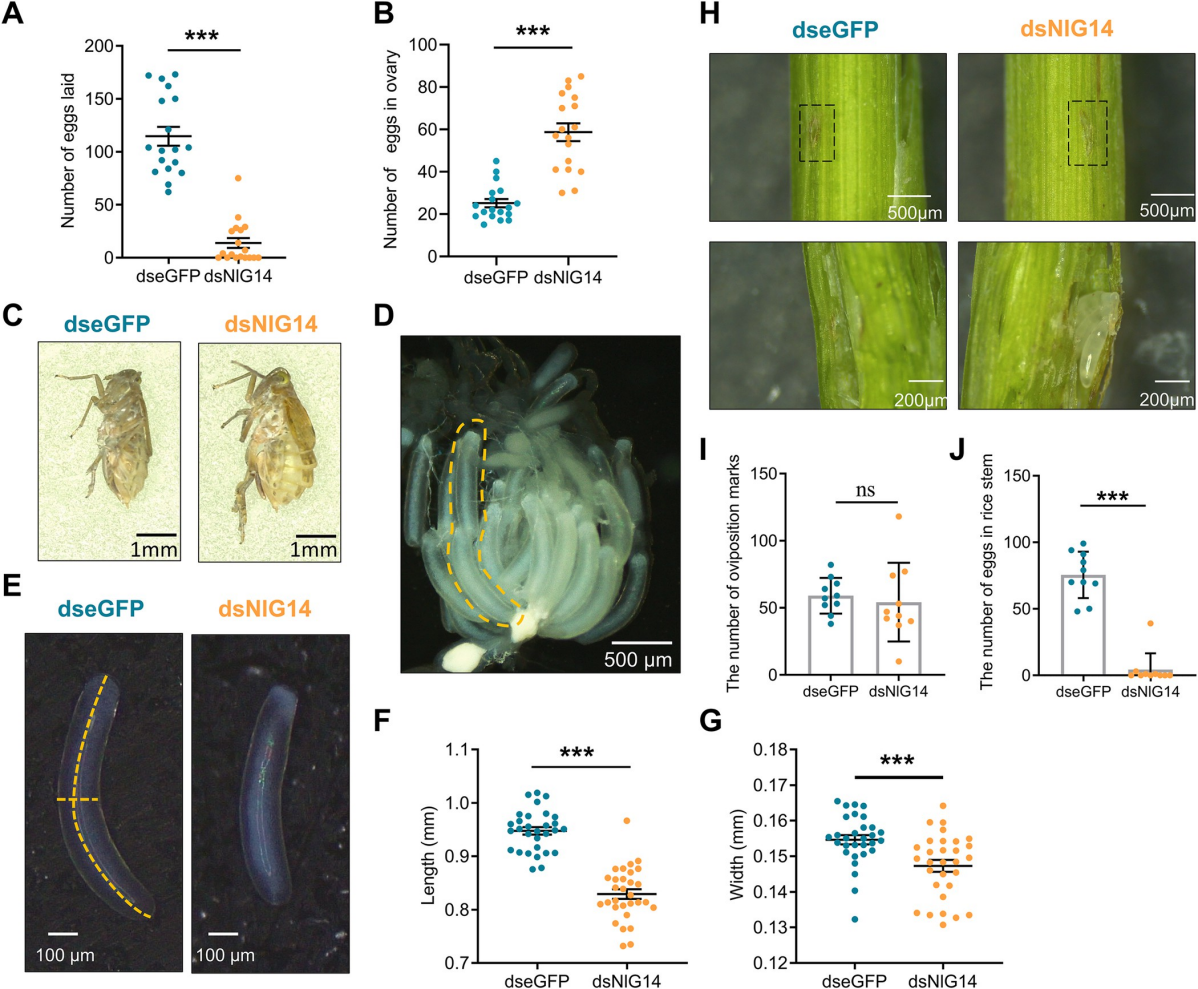
NlG14 knockdown caused the ovulation deficiency in BPH. (A, B) The number of eggs in rice stem laid by BPH females (A) and the number of eggs remained in the ovaries (B) within 7 DPE. A female injected with dsNlG14 or dseGFP was paired with an untreated male for mating. Data are mean±SE (n = 20). (C)Representative images of females injected with dsNlG14 or dseGFP mated with untreated males. (D) Eggs in ovarioles. Two mature eggs were retained one ovarian tubule of some dsNlG14-treated females. (E) Comparison of mature egg shapes between dsNlG14- and dseGFP-treated females. (F, G) Quantification of the length (F) and width (G) of eggs shown in E. Data are mean ±SE (n = 30). (H) Image of oviposition marks before and after dissection. The black box shows the oviposition marks. Below is an enlarged image of the oviposition marks. (I, J) The number of oviposition marks (I) and the number of eggs (J) in rice stem within 7 DPE to 10 DPE. Data are mean ±SE (n = 10). Significant differences were determined using Student’ s t-test: ***P<0.001, ns, no significance.

To further verify the effect of NlG14 knockdown on BPH reproduction, we performed a fecundity experiment. As expected, the number of eggs laid in the rice stem was significantly reduced, and the dissection of the abdominal ovaries from BPH females treated by dsNlG14 showed the obvious accumulation of mature eggs in the ovary (Fig 3A–3C).

Upon further observation of the ovaries, two mature eggs were accumulated in one ovarian tubule of BPH females treated by dsNlG14, which was hardly seen in normal ovaries as in control females treated by dseGFP (Fig 3D). In addition, the length and width of eggs in the ovaries of the treatment group were reduced by 12.6% and 5.2%, respectively (Fig 3E–3G). Lipids are important energy sources and are associated with egg development and successful reproduction [21–23]. We measured the contents of triglyceride (TAG) and glyceride of eggs in the ovaries. The results showed no significant difference in TAG and glyceride content per mg protein in dsNlG14- and dseGFP-treated eggs (S1 Fig).

When laying eggs, BPHs insert its hard ovipositor into rice stem and leave distinct oviposition marks on the stem surface. To clarify whether the abnormal egg-laying behavior contributed to abnormal oviposition, we analyzed the number of oviposition marks and eggs in rice stems. Compared to the control, the oviposition of the female injected with dsNlG14 decreased, but the number of oviposition marks had no significant differences (Fig 3H–3J).

### Knockdown of NlG14 resulted into salivary gland malformation

**Fig 4:**
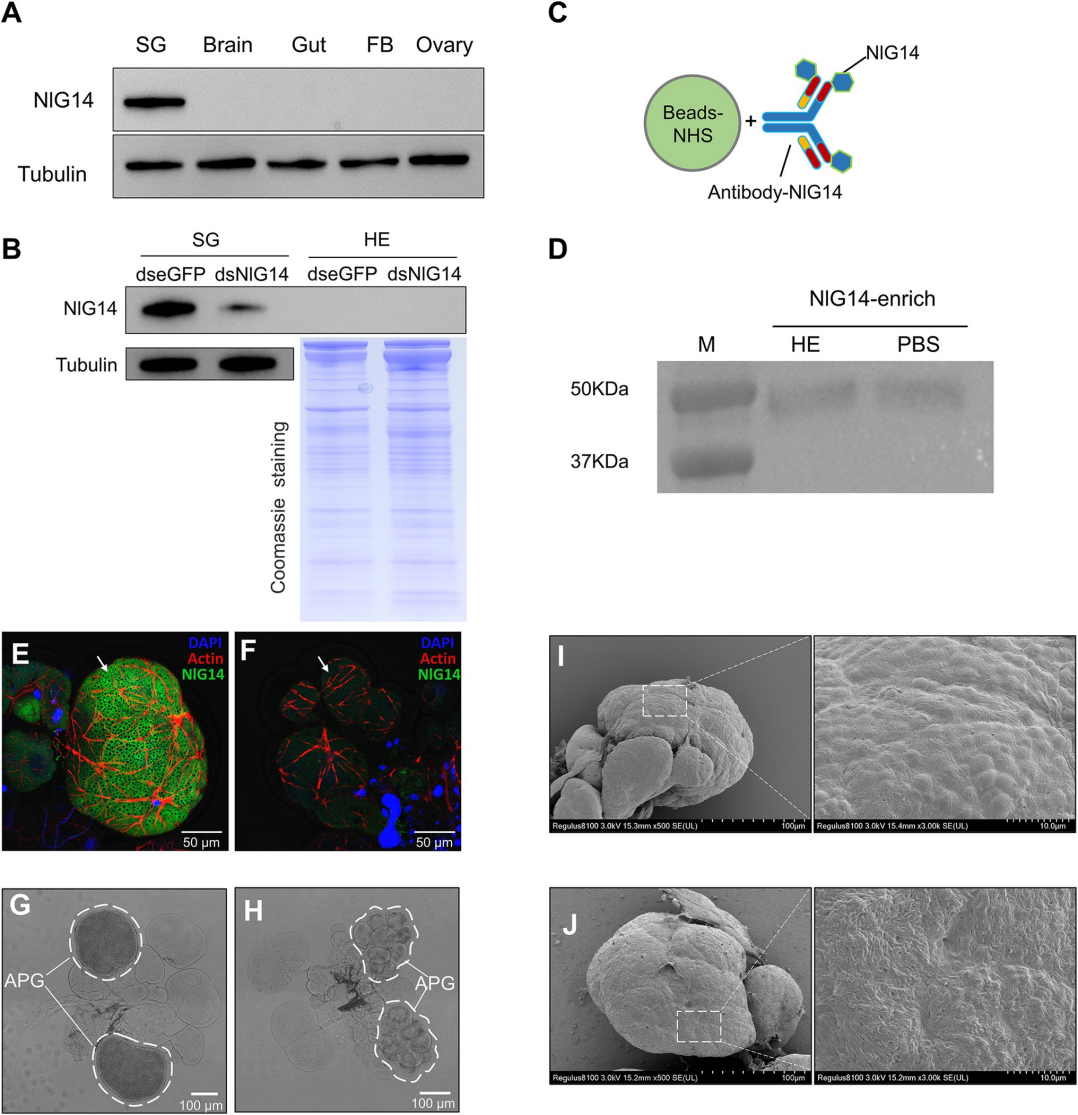
Salivary gland-derived NlG14 were not secreted into BPH hemolymph. (A) NlG14 protein detection in multiple tissues was analyzed using western blotting. FB, Fat body; SG, Salivary gland. Tubulin was used as the loading controls. (B) Immunoblotting of NlG14 in salivary glands and hemolymph. Tubulin and Coomassie staining were used as the loading controls for salivary gland and hemolymph, respectively. (C) Schematic representation of NlG14 antibody covalently coupled to NHS-activated magarose beads. (D) Enrichment analysis of NlG14 in hemolymph. (E, F) The fluorescence brightness of NlG14 protein was significantly reduced in APG (A-follicle of the principal gland) of BPH injected with dsNlG14 (F) when compared to the control BPH injected with dseGFP (E). (G, H) Salivary gland images of control (G) and treatment (H) BPH females. (I, J) SEM images of salivary glands from control (I) and treatment (J) BPH females. The right was an enlarged view of the white box in the left image.

It has recently been shown in *Drosophila* that the salivary gland-derived secreted proteins could regulate insect growth and development through the endocrine system [7]. Whether could NlG14 be secreted into the hemolymph and directly act on BPH ovary? To address this question, NlG14 protein in different tissues was determined. The results showed that NlG14 was only detected in the salivary gland (Fig 4A). To validate the presence of NlG14 in hemolymph, the western blot analysis was performed. The result showed that the content of NlG14 protein in salivary glands of BPH females injected with dsNlG14 was significantly lower than that in the control injected with dseGFP (Fig 4B). However, in the hemolymph samples, NlG14 was not detected in either the treatment or the control (Fig 4B).

To further rule out the possibility that the non-detection was attributed to low NlG14 secretion, we enriched the potential NlG14 in hemolymph using NHS-activated magarose beads coupled to NlG14 antibody (Fig 4C). Firstly, we enriched NlG14 from the whole female body to test the bead device. The results demonstrated that the anti-NlG14 beads successfully enriched a large amount of NlG14, and the absence of corresponding bands in the PBS negative control lane also indicated that the antibodies to the beads were not significantly shed (S2 Fig). The anti-NlG14 beads were then used to enrich potential NlG14 protein in hemolymph samples. The immunoblotting results showed no significant difference in the bands between the hemolymph and the PBS negative control samples (Fig 4D). The faint bands shown in both lanes were presumed to be heavy chains of antibody. These results revealed that NlG14 could not be delivered to the hemolymph of BPH through an endocrine mechanism, which indicated that NlG14 might indirectly act on the ovaries of BPH.

To explore the potential effects of NlG14 knockdown on salivary glands, the immunostaining and scanning electron microscopy were performed. As expected, immunostaining results showed that the green fluorescence signal of salivary glands with NlG14 knockdown was significantly reduced (Fig 4E and 4F). The APG crumpled after NlG14 reduction (Fig 4G and 4H). By scanning electron microscopy, BPH APG surface was smooth and had many mamelons, while the surface of APG BPH with NlG14 knockdown became rough and the mamelons were not obvious (Fig 4I and 4J). These results showed that NlG14 knockdown deformed the salivary gland APG of BPH, which might affect the normal secretory function of the salivary gland.

### KEGG enrichment analysis and validation of transcriptome

**Fig 5:**
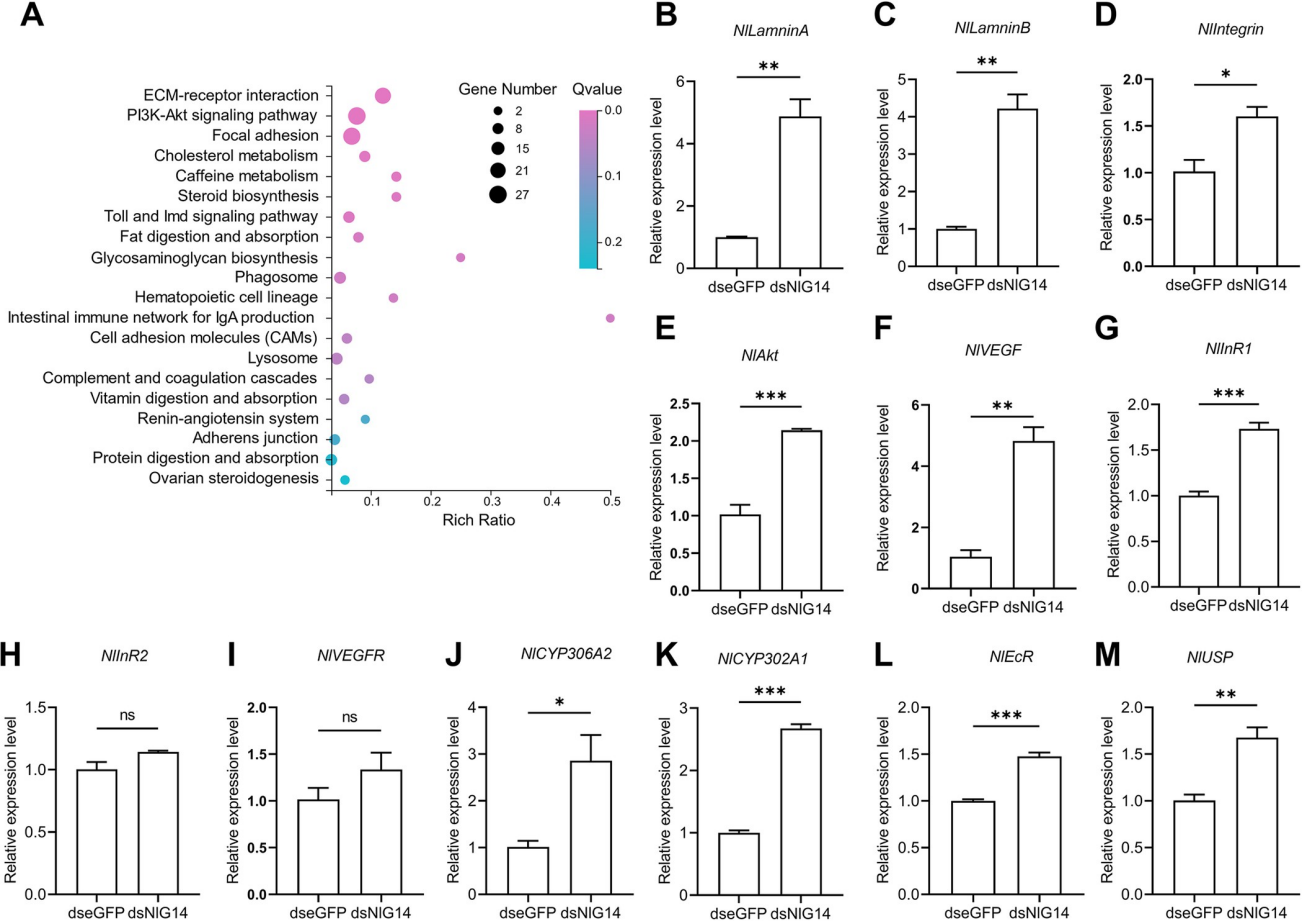
KEGG pathway enrichment analysis and quantitative verification. (A) KEGG pathway enrichment analysis of differentially expressed genes with the up-regulated expression levels in ovaries of BPH with NlG14 knockdown when compared to that of control. (B-M) RT-qPCR validation of expression levels of select genes in important pathways from KEGG analysis. Data are mean±SE (n = 3). Significant differences were determined using Student’ s *t*-test: *P<0.05; **P<0.01; ***P<0.001; ns, no significance.

To further elucidate the effects of NlG14 knockdown on the ovary, we performed transcriptome sequencing of ovaries from BPH females of dsNlG14 treatment and dseGFP control. KEGG pathway enrichment of up-regulated genes covered some important pathways, such as ECM-receptor interaction (ko04512), PI3K-Akt signaling pathway (ko04151) and Steroid biosynthesis (ko00100) (Fig 5A).

RT-qPCR was performed to validate the significant up-regulation of genes from these pathways. From ECM-receptor interaction, the expression of the three genes of laminins and its receptor integrin were significantly increased in BPH with NlG14 knockdown (Fig 5B–5D). In PI3K-Akt signaling pathway, the up-regulation was observed for *NlAkt*, vascular endothelial growth factor (*NlVEGF*) and insulin receptor (InR) genes *NlInR1*, but not *NlInR2*. (Fig 5E–5H). The gene of the vascular endothelial growth factor receptor (*NlVEGFR*), a receptor tyrosine kinase (RTK), was also up-regulated, although there was no significant difference (Fig 5I). RT-qPCR results also showed that two genes from the ecdysone synthesis and two genes of the ecdysone receptor were expressed at the significantly higher levels in the ovary of BPH with NlG14 knockdown than that of the control (Fig 5J–5M).

### Knockdown of NlG14 enhanced the expression of two insulin-like genes in the brain and an insulin receptor gene in ovary

**Fig 6:**
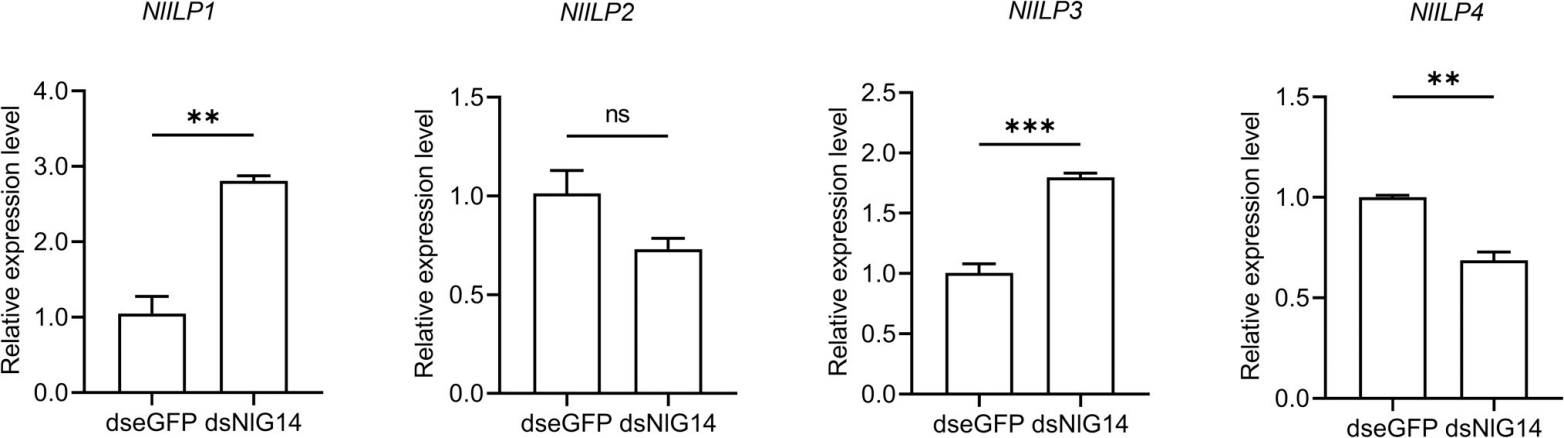
Gene expression of insulin-like peptides 1–4 (*NlILP1*-*4*) in brain of BPH with NlG14 knockdown or control. Data are mean±SE (n = 3). Significant differences were determined using Student’ s t-test: **P<0.01; ***P<0.001; ns, no significance.

Blood feeding triggered the brain to release ovary ecdysteroidogenic hormone (OEH), ILP3 and ILP4 in female mosquitoes, in which ILP3 signal activated insulin signaling and Akt pathway to stimulate synthesis of ecdysteroids by binding receptor MIR [24,25]. We raised a question whether NlG14 could affect the secretion of insulin-like peptides (ILPs) in BPH brain, which then regulated ovary development through ecdysteroid biosynthesis, as indicated by KEGG analysis results. RT-qPCR was performed on ILPs genes in brain tissue of BPH. The results showed that *NlILP1* and *NlILP3* were significantly up-regulated in the brain of BPH treated by dsNlG14 when compared to dseGFP control, while *NlIlp4* were down-regulated (Fig 6). Previous studies have shown that *NlILP1* and *NlILP3* had the highest expression levels in the head, with NlILP3 acting as the major insulin ligand for NlInR1 leading to the long-winged variant [26]. Here we also found that *NlInR1* was expressed at a significantly high level in the ovary of BPH with NlG14 knockdown than that of control BPH (Fig 5G). These results suggested that NlG14 knockdown might regulate the secretion of NlILP1 and NlILP3 in brain and the expression of *NlInR1* in ovary, whose interaction subsequently regulated the ecdysteroid biosynthesis through the insulin-PI3K-Akt signaling pathway, as indicated by results from KEGG analysis (Fig 5A).

## Discussion

The glands are usually divided into endocrine and exocrine glands. Exocrine glands release their products through ducts into the surface of the target cells or organs, and endocrine glands release substances directly into the general circulation. Traditionally, the insect salivary glands are typically exocrine glands that help them feed on plants. For example, piercing-sucking herbivores secrete saliva into plants to regulate the plant’s defense response when feeding [27,28]. Some mammalian glands have both endocrine and exocrine functions, such as the pancreas and kidney. Recently, evidence for the endocrine function of salivary glands has been provided in *Drosophila* [7]. In the present study on BPH, although the salivary protein NlG14 could not be secreted into the hemolymph, its knockdown might indirectly inhibit ovarian ovulation by affecting the normal endocrine function of salivary glands.

Contrary to our expectation, we found that knockdown of NlG14 did not delay systemic development and ovarian of BPH, but resulted into the enlarged ovaries due to the accumulation of mature eggs. We previously demonstrated that NlG14 was highly expressed in salivary glands at the mRNA level [13]. In this study, it was confirmed that NlG14 was localized only in salivary glands and could not be secreted into hemolymph at the protein level. We firstly observed the surface of APG of salivary gland by scanning electron microscopy. APG has a smooth surface and many mamelons, and these structures may be related to potential endocrine functions. The reduction of NlG14 protein caused the surface of APG rough and the mammary process was not obvious. These tissue lesions might affect the normal secretion function of APG.

KEGG pathway enrichment of up-regulated genes showed that some pathways such as ECM-receptor interaction, PI3K-Akt signaling pathway and steroid biosynthesis were significantly enriched. ECM-receptor interaction is a micro-environmental pathway that maintains cell and tissue structure and function [29]. A complex mixture of structure and function makes up the ECM, including collagen, fibronectin (FN), and laminin. Laminin is involved in the assembly of the ECM of ovarian muscle, which is indispensable for ovarian muscle contraction [17,30,31]. Cell membrane receptors play a key role in cell-ECM interactions, such as integrins [32,33]. Nllaminin and Nlintegrin were significantly upregulated in BPH ovaries (Fig 5B–5D). In addition, immunofluorescence staining of the lateral oviduct showed that NlG14 knockdown significantly affected the contraction of the muscles of lateral oviduct (Fig 2D). These results suggested that NlG14 knockdown affected the contraction of the muscles of lateral oviduct and displacement of the LOSC by indirectly affecting insulin and ECM-receptor interaction signaling pathway. The oviduct secreted protein (Nlodsp) of BPH is one of the LOSCs involved in the delivery of eggs from the lateral oviduct to the common oviduct [18]. The RT-qPCR results showed that the expression level of *Nlodsp* in ovaries of BPH injected with dsNlG14 was only 25% of that in the control (S3 Fig). This result indicated that the expression of oviduct secretory protein gene was suppressed, which might affect its function of egg transport.

The PI3K-Akt signaling pathway is a widely studied intracellular signaling that responds to a variety of extracellular signals such as ECM, VEGF, insulin, thus promoting metabolism, proliferation, growth, angiogenesis, etc. [34]. Insulin also could induce the expression of laminin and VEGF [35,36]. BPH possesses four ILP genes and two insulin receptor genes, all of which play important roles in female fecundity [37,38]. Akt-interacting protein (AKTIP) was highly expressed in gravid female, and RNAi of *AKTIP* seriously affected ovarian development of female BPH [39]. In this study, knockdown of NlG14 caused the up-regulation of insulin-pi3k-akt signaling probably due to the overexpression of *NlILP1* and *NlILP3* in brain and *NlInR1* in ovary. In *Aedes aegypti*, ILP3 produced in the brain binded to MIR in the ovary, and then activated insulin and Akt signaling to promote ecdysteroid biosynthesis [24,25]. Here it was deduced that silencing NlG14 upregulated two insulin-like genes in the brain and an insulin receptor gene in ovary, and high insulin increased the expression of Nllaminin and Nlintegrin genes. Higher Nllaminin and Nlintegrin caused the abnormal contraction of lateral oviduct muscle and then the displacement of LOSC. Finally, the displacement of LOSC disrupted the ovulation of BPH females.

The 20E signal in insect ovaries is critical for ovulation. For example, 20E produced in follicle cells in *Drosophila* caused response of mature follicles to neuronal stimulation of ovulation by activating ECR.B2 [14]. The ovary is an important physiological tissue for the production of ecdysteroid in female adult BPH, and the knockdown of most ecdysteroid biosynthetic enzymes genes disturbed ovarian development [40]. To further confirm the effect of hormonal signaling on the ovaries, we examined the expression levels of juvenile hormone and ecdysone related genes in whole body on 1, 4, 7 and 10 day post-eclosion (DPE), as well as in the ovaries on 7 DPE. Results showed that there was no significant difference in the expression levels of ecdysin synthesis genes and receptor gene at the whole body level, except for CYP306A2, between BPH with *NlG14* knockdown and the control (S4 Fig). However, almost all genes in ecdysone synthesis and receptors were expressed at significantly higher levels in the ovary of BPH with *NlG14* knockdown than that of the control (S4 Fig). The *NlJHAMT*, a key synthetic gene of juvenile hormone, and *NlMET*, a receptor gene of juvenile hormone, did not show significant differences in expression of mRNA (S5 Fig). These results suggest that down-regulation of *NlG14* significantly induced the expression of genes in ecdysone synthesis pathway and receptors in the ovary, but not at the systemic level.

Taken together, the complex signaling and hormone upregulation disorder revealed by transcriptomic data might cause displacement of LOSC in BPH, leading to ovulation disorders, revealing the coupled role of salivary gland and brain in regulating insect ovulation.

The salivary gland of BPH contained a potential large effector repertoire [20] which was thought to be involved in complex insect-plant interactions. However, given the example of the endocrine function of insect salivary gland [7], some of these effector might regulate its growth and development through endocrine. The specific endocrine factors affected by NlG14 on salivary gland endocrine function have not been identified and need to be further studied.

**Fig 7:**
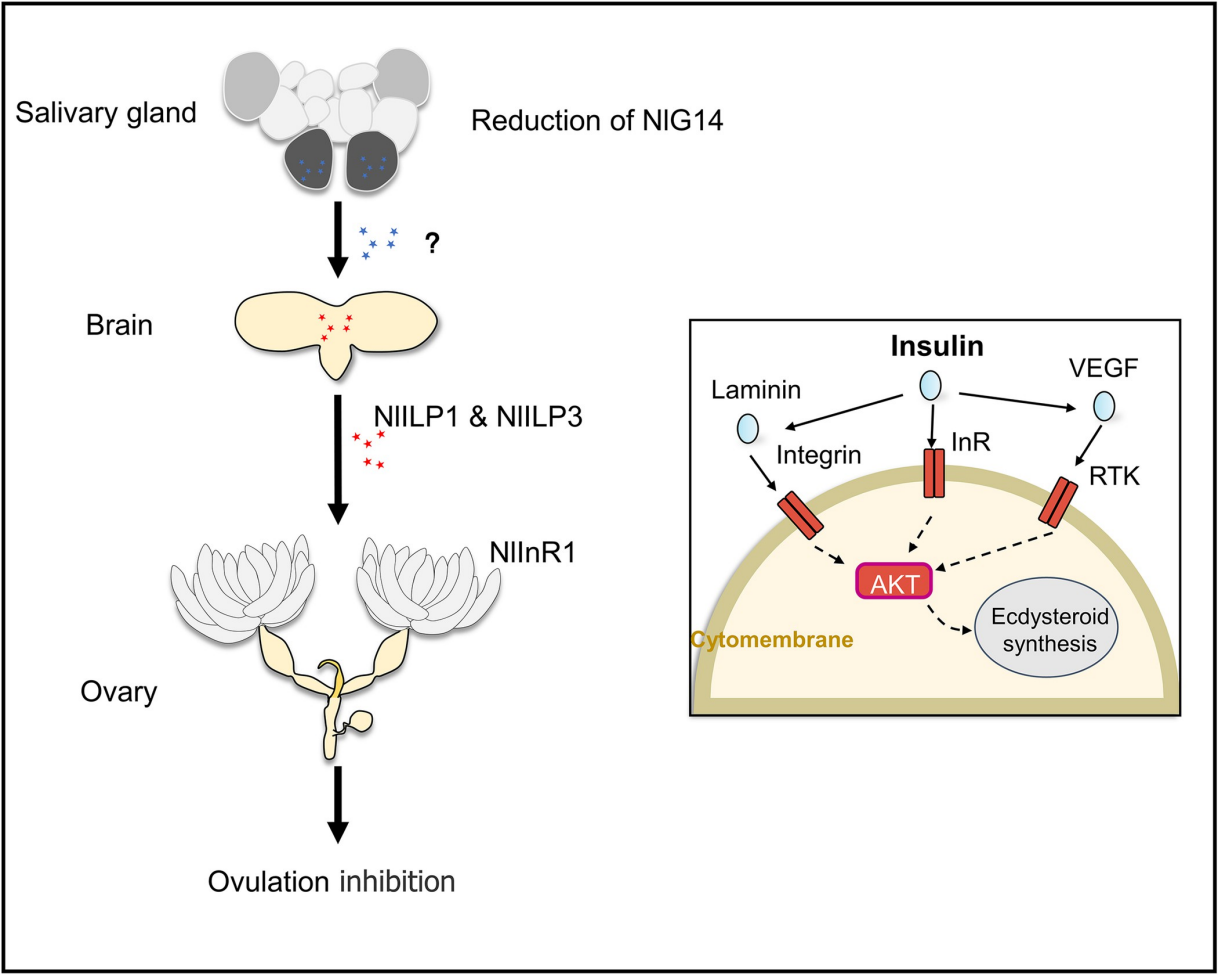
Model of the molecular regulation of NlG14 on ovarian ovulation in BPH.

Based on these results, we proposed a model of the molecular regulation (Fig 7): Reduction of NlG14 affected the endocrine function of salivary glands in BPH. Abnormalities of unknown endocrine factors promoted the secretion of insulin-like peptides NlILP1 and NlILP3 in brain, which bound to NlInR1 and then led to the overexpression of insulin-PI3K-Akt signaling and ecdysone in the ovary. Complex signaling and hormonal disturbances resulted into the displacement of LOSC, which then inhibited BPH ovulation.

## Materials and methods

### Insects and plant materials

BPHs were originally collected from Hangzhou, Zhejiang province, southeast China. Insects were then maintained indoors with fresh Xiushui 11 variety rice seedlings under standard conditions of 27 ± 1°C and 70% ± 10% relative humidity with a 16 h:8 h (light: dark) photoperiod, without exposure to any pesticides.

### RNA interference

RNA interference experiments were performed as previously described [13]. Briefly, NlG14 and enhanced green fluorescent protein (eGFP) fragments were amplified by specific primers containing the T7 promoter. The dsRNAs were synthesized using the T7 RiboMAX Express RNAi System (Promega, Madison, WI, USA) according to the manufacturer’s instructions. Fifty nanoliter of dsRNAs at 3 mg/mL were injected into 5th instar BPH nymphs using Nanoliter-2010 microinjector (World Precision Instruments, Sarasota, FL).

### Developmental analysis of the ovary

Insect within 24 h of eclosion were defined as 1 day post-eclosion (DPE). One-DPE female adults was transferred to plastic cups containing rice seedlings. The number of eggs in the ovaries was counted and photographed with Leica S9i (Leica Microsystems, Germany) on the 1, 4, 7 and 10 DPE.

### Fecundity analysis

One 1 DPE female adult injected with dseGFP or dsNlG14 and one 1 DPE untreated male were placed in glass tubes containing 5 rice seedlings. The number of eggs in ovaries and in the stem of rice were counted, respectively, on the 7 DPE under a microscope.

### Oviposition marks analysis

A 7 DPE virgin female was put into a glass tube containing 5 rice seedlings. The number of oviposition marks on the rice and the number of eggs in the rice stem were counted and photographed on the 10 DPE under a microscope.

### Egg size measurements

Ovaries were dissected from the 7 DPE virgin females and photographed with Leica S9i (Leica Microsystems, Germany). The length and width of retained mature eggs were measured using AutoCAD 2017 software (Autodesk Inc., San Rafael, CA, USA). Because the eggs are "banana" shaped, the length was defined as the curve between the two distant ends and the width was the distance of the midpoint of the curve.

### TAG and glyceride assays

Eggs TAG and glyceride contents were quantified as described previously [41]. About 60 eggs were homogenized in 200 μL PBST (pH 7.4, 0.5% Tween-20). The protein concentration of samples was detected using an Easy II Protein Quantitative Kit (BCA) (TransGen, Beijing, China). The 40 μL homogenate was incubated at 70°C for 5 min and then incubated with either 40 μL PBS or Triglyceride Reagent (Sigma) at 37°C for 30 min and centrifuged (13,200 × g, 4°C, 5 min). A 60 μL sample was incubated with 200 μL Free Glyceride Reagent (Sigma) for 5 min at 37°C in 96-well plate. TAG and glyceride concentrations were measured at 540nm using a microplate reader (VERSA max, Molecular Devices, USA)

### SDS-PAGE analysis and protein identification

Deposits in bursa copulatrix (DBC), lateral oviduct secreted components (LOSC), and eggs were dissected and homogenized in phosphate-buffered saline (PBS) buffer contain 1×protease inhibitor cocktail. The homogenate was mixed with loading buffer, denatured and centrifuged. Supernatants were separated on SDS–PAGE gels (GenScript, Nanjing, China). The protein samples sent to the Key Laboratory of Bio-interactions and Agricultural Pest Management in Nanjing Agricultural University for tandem mass spectrometry analysis on Orbitrap Exploris 480 Mass Spectrometers (Thermo Fisher Scientific).

### Quantitative RT-PCR (RT-qPCR)

Total RNA was extracted from female adult tissues by using Trizol reagent (Invitrogen). One microgram of total RNA was used for cDNA synthesis with HiScript III RT SuperMix for qPCR (Vazyme, Nanjing, China) according to the manufacturer’s instructions. RT-qPCR was performed with SYBR qPCR Master Mix (Vazyme, Nanjing, China) using a QuantStudio Real-Time PCR System (Applied Biosystems). Glyceraldehyde-3-phosphate dehydrogenase (GAPDH) and ribosomal protein S15 (RPS15) were used as the reference genes. The relative mRNA expression levels were calculated using the 2^-ΔΔCT^ method [42]. All experiments were independently performed with three biological replicates. All primers used for RT-qPCR are listed in S1 Table.

### Protein extraction and immunoblot analysis

Salivary gland, brain, gut, fat body and ovary proteins from female adults were extracted and homogenized in 1 mL PBS. The extract was centrifuged at 13,000 × g for 15 min at 4°C and the supernatant was collected. To prepare the hemolymph sample, total hemolymph of about 100 nymphs was collected by tearing their integument carefully in 20 μL PBS buffer containing 1x proteinase inhibitor cocktail. After centrifugation, the cell-free supernatants were further concentrated to a final volume of 500 μL using a 10 kDa ultrafiltration tube.

Protein samples were mixed with loading buffer, denatured, and separated on 4%-20% SDS–PAGE gels (GenScript, Nanjing, China). The antibodies used in the experiments included rabbit anti-NlG14 (1:5,000) custom-developed by Zoonbio (Nanjing, China), mouse anti-tubulin (1:7,500; Beyotime), HRP-conjugated goat anti-rabbit (1:10,000; Cwbio), and HRP-conjugated goat anti-mouse (1,10, 000; Cwbio). Tubulin was used to estimate the protein loading. Coomassie staining-based total proteins were used as the loading control in analyzing the proteins in the hemolymph.

### NlG14 protein enrichment

NHS-activated magarose beads effectively reduce antibody drop and reduce interference caused by antibody heavy and light chains in Western blot compared to protein A/G-agarose beads. NlG14 antibody was covalently coupled to NHS-activated magarose beads (Smart-Lifesciences) according to the instructions. The effect of antibody coupling was detected by the enrichment of NlG14 of whole BPHs. In brief, 0.5 g BPHs were homogenized in 2 mL PBS containing 1 x protease inhibitor cocktail and centrifuged at 13,000 × g for 15 min at 4°C to collect cell lysates. Cell lysate protein concentrations were determined by BCA assay and then diluted to 10 mg/mL in PBS containing 1 x protease inhibitor cocktail. One milliliter cell lysate was added into 50 μL pretreated beads, and 1 mL PBS was added into the control beads for 30 min. After washing the impurity protein, the beads were treated with 200 μL of 0.1 M glycine (pH 3.0) to collect the eluent. Immediately after that, 20 μL 1M Tris-HCl was added to neutralize pH. The samples were boiled in loading buffer and tested with SDS-PAGE.

For the enrichment of NlG14 in hemolymph, 1 mL hemolymph sample was prepared according to the abovementioned process. Beads were used for enrichment, and PBS was used as control. The enriched protein samples were added to loading buffer and boiled for western blot detection.

### Immunostaining

Immunostaining was performed as previously described [13]. Samples were dissected in PBS and fixed in 4% paraformaldehyde overnight at 4°C. After washed three times with PBS, salivary glands were incubated in 2% TritonX-100 at room temperature for 30 min. The following primary antibody anti-NlG14 (1:200) were used. After washing in PBS, the samples were incubated with anti-rabbit IgG (whole molecule)-FITC antibody produced in goat (1:200, Sigma-Aldrich), DAPI (1:10, VECTASHIELD Vector Laboratories), and Actin-Tracker Red-Rhodamine (1:200, Beyotime) or Actin-Tracker Green (1:200, Beyotime) at room temperature for 1 h. After washing with PBS, samples were mounted in Antifade Mounting Medium (Beyotime) and observed using a Leica TCS SP8 confocal laser-scanning microscope. The wavelengths for DAPI were excitation at 405 nm and emission at 430–460 nm, for FITC excitation at 488 nm and emission at 500–530 nm, and for Rhodamine excitation at 561 nm and emission at 570–600 nm.

### Scanning electron microscopy (SEM) analysis

Salivary glands of 7 DPE BPH were dissected in PBS buffer and immediately fixed in 20% glutaraldehyde. The samples were then dehydrated in a gradually increasing series of ethyl alcohol (50%, 70%, 80%, 90%, 100%). After dried in a critical-point dryer, the samples were coated with gold-palladium for further observation. Scanning electron microscopy (SU8100, HITACHI, Japan) was used to characterize the morphology of samples.

### Transcriptomic sequencing

Ovaries of 7 DPE BPHs were collected and immediately placed in RNAlater (Invitrogen). After removing RNAlater by centrifugation and liquid nitrogen quick freezing, the tissues were stored at −80 °C. Total RNA was extracted using Trizol reagent (Invitrogen). The RNA quality and quantity were determined using a Nanodrop ND1000 spectrophotometer (NanoDrop Technologies, Wilmington, DE, USA) and an Agilent Bioanalyzer 2100 (Agilent, Palo Alto, CA, USA) with an Agilent RNA 6000 Nano Kit. The A260/280 ratio was used to check for protein contamination and RNA integrity was assessed by running the samples on a 1% denaturing agarose gel. High-quality RNA was utilized for library construction and sequenced on the BGISEQ-500 platform (BGI, Wuhan, China) with 150 bp paired-end reads. The DESeq2 software [43] was used to identify differentially expressed genes (DEGs). The threshold values log2foldchange (FC) >1 and Qvalue < 0.05 indicated up-regulated DEGs. KEGG pathway enrichment analysis of up-regulated DEGs was performed utilizing the KEGG pathway database (http://www.genome.jp/kegg).

The raw data of this project are shown in the S3 Table.

## Supporting information

S1 Fig Effects of NlG14 knockdown on lipid mobilization of eggs. TAG (triacylglycerol) (A) and glyceride (B) contents were determined using eggs in ovary of virgin females injected with dsNlG14 or dseGFP on 7 DPE. Data are mean±SE (n = 3). Significant differences were determined using Student’ s *t*-test: ns, no significance.
(TIF)

S2 Fig Enrichment of NlG14 protein by NHS-activated magarose beads coupled to NlG14 antibody in whole female body of BPH. Asterisk indicate the target band of the protein.
(TIF)

S3 Fig NlG14 knockdown caused changes in the oviduct secreted protein (Nlodsp) gene. The *Nlodsp* genes expression variations of whole body on 1, 4, 7, 10 DPE and ovary on 7 DPE. Data are mean±SE (n = 3). Ov, ovary. Significant differences were determined using Student’ s *t*-test: *P<0.05; ***P<0.001.
(TIF)

S4 Fig NlG14 knockdown caused changes in ecdysteroid biosynthetic pathway and receptor genes. (A) Schematic sketch of ecdysteroid biosynthetic pathway in BPH. (B-I) The genes expression variations of whole body on 1, 4, 7, 10 DPE and ovary on 7 DPE. Data are mean±SE (n = 3). Ov, ovary. Significant differences were determined using Student’ s *t*-test: *P<0.05; **P<0.01; ***P<0.001.
(TIF)

S5 Fig NlG14 knockdown caused changes in juvenile hormone biosynthetic and receptor genes. (A-B) The juvenile hormone biosynthetic (A) and receptor (B) genes expression variations of whole body on 1, 4, 7, 10 DPE and ovary on 7 DPE. Data are mean±SE (n = 3). Ov, ovary. Significant differences were determined using Student’ s *t*-test: **P<0.01.
(TIF)

S1 Table Gene specific primers in this study. (XLSX)

S2 Table The LC-MS results of the DBC and LOSC. (XLSX)

S3 Table Raw data of this project. (XLSX)
