# Supplementary figures and images for: Knockdown of the salivary protein gene NlG14 caused displacement of the lateral oviduct secreted components and inhibited ovulation in Nilaparvata lugens
Source: PLoS Genet. 2023 Apr 3;19(4):e1010704. doi: 10.1371/journal.pgen.1010704 (PMC10101634; doi:10.1371/journal.pgen.1010704)

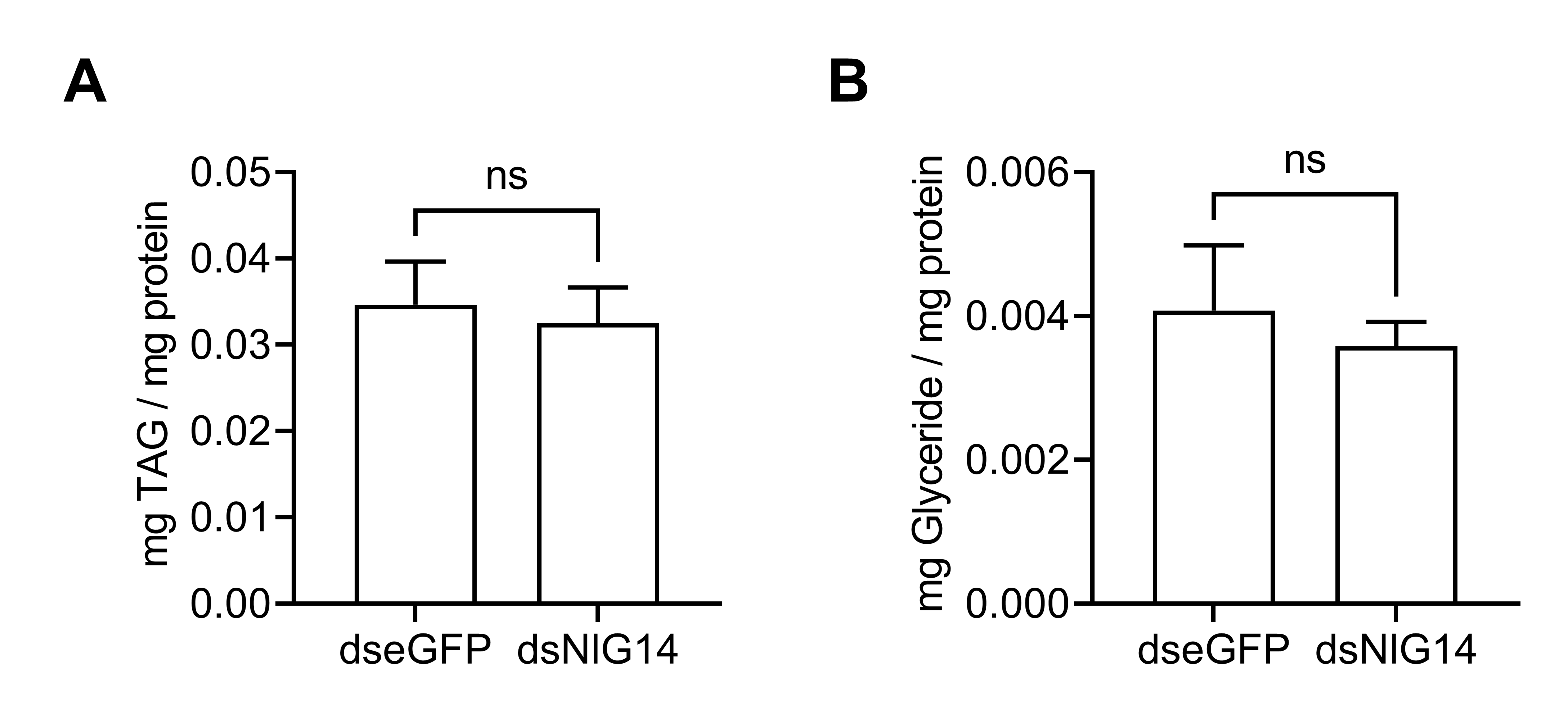

Supplement: S1 Fig — TAG (triacylglycerol) (A) and glyceride (B) contents were determined using eggs in ovary of virgin females injected with dsNlG14 or dseGFP on 7 DPE. Data are mean±SE (n = 3). Significant differences were determined using Student’ s t-test: ns, no significance. (TIF) [file pgen.1010704.s001.tif]

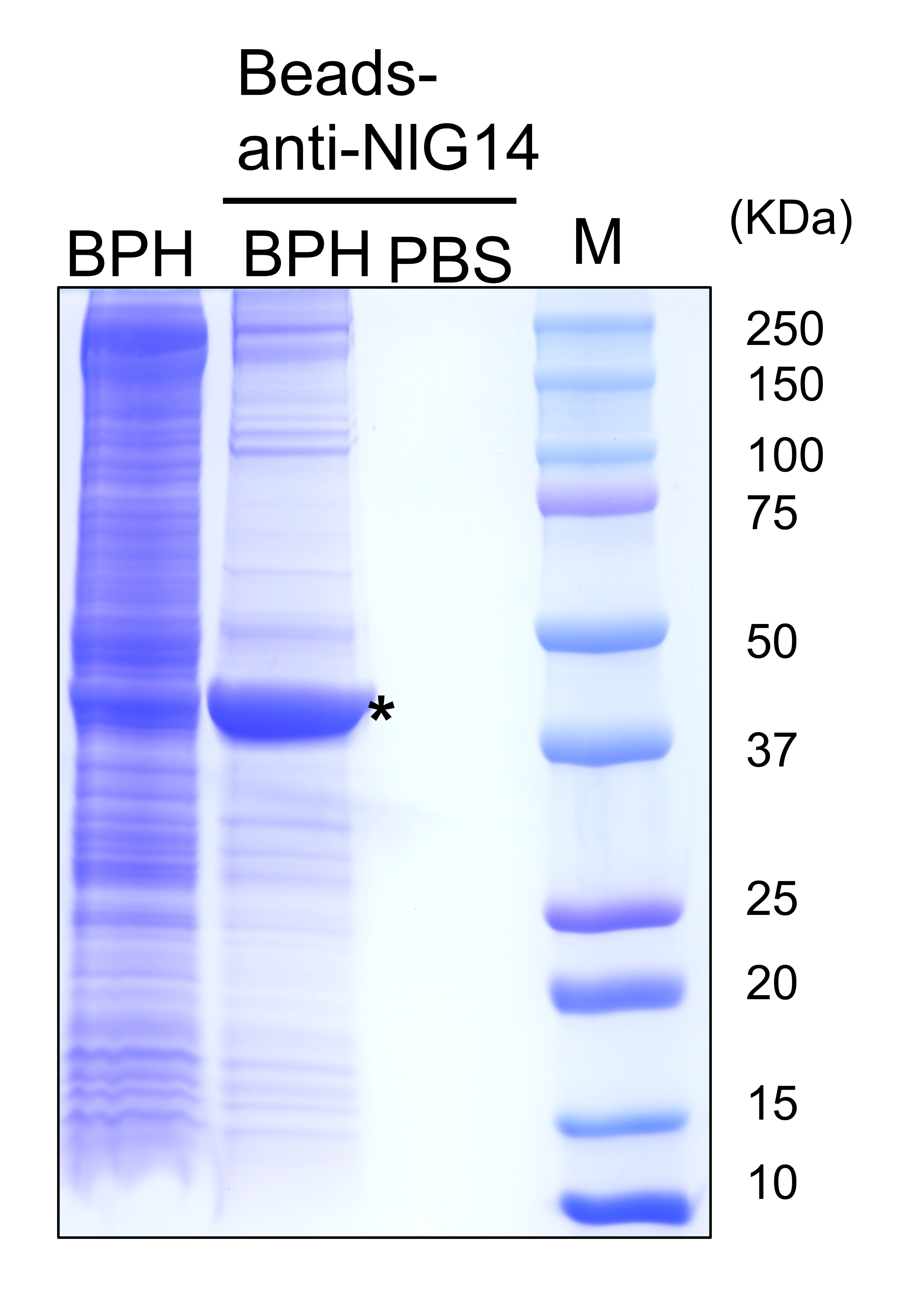

Supplement: S2 Fig — Asterisk indicate the target band of the protein. (TIF) [file pgen.1010704.s002.tif]

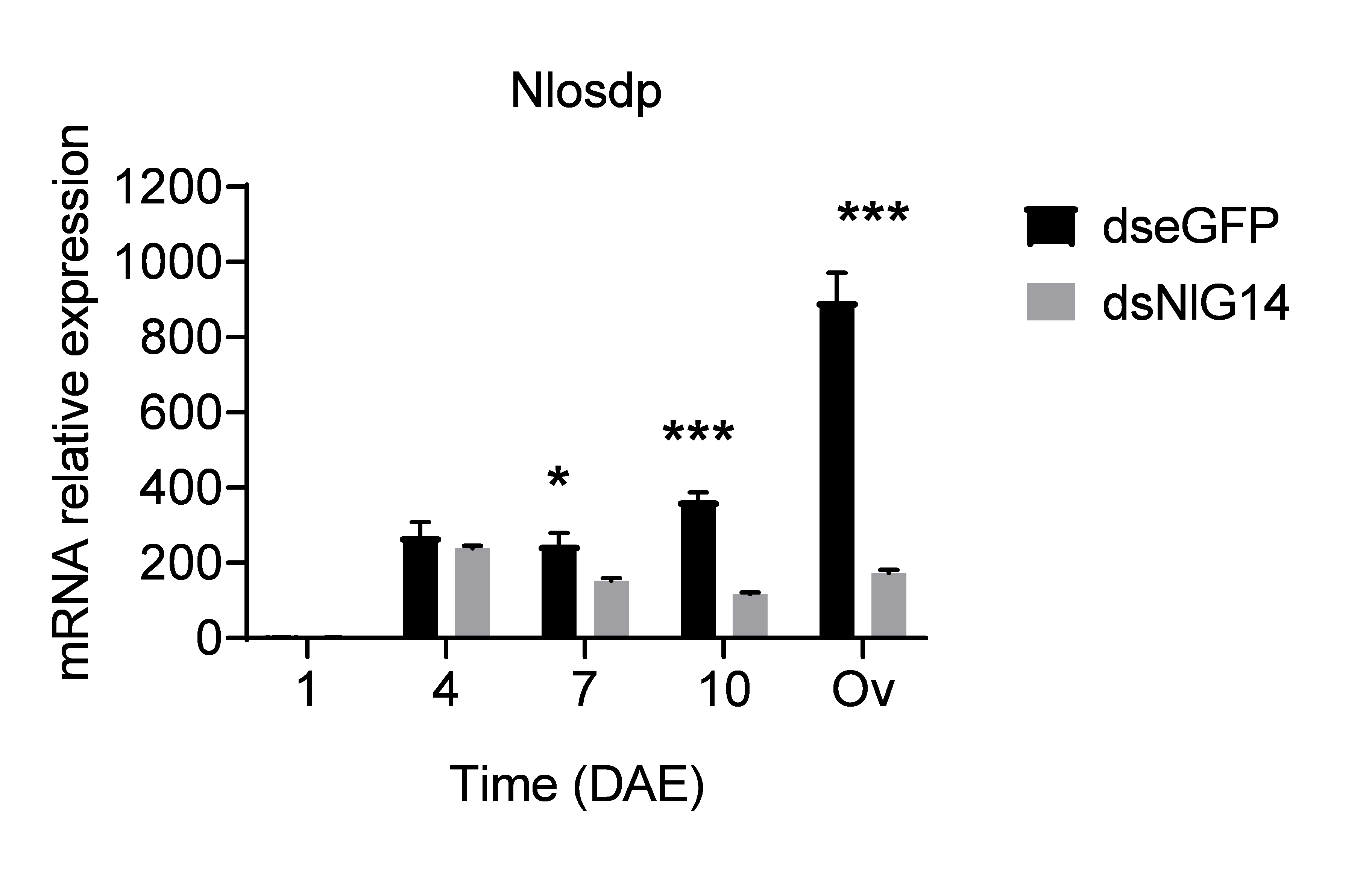

Supplement: S3 Fig — The Nlodsp genes expression variations of whole body on 1, 4, 7, 10 DPE and ovary on 7 DPE. Data are mean±SE (n = 3). Ov, ovary. Significant differences were determined using Student’ s t-test: *P<0.05; ***P<0.001. (TIF) [file pgen.1010704.s003.tif]

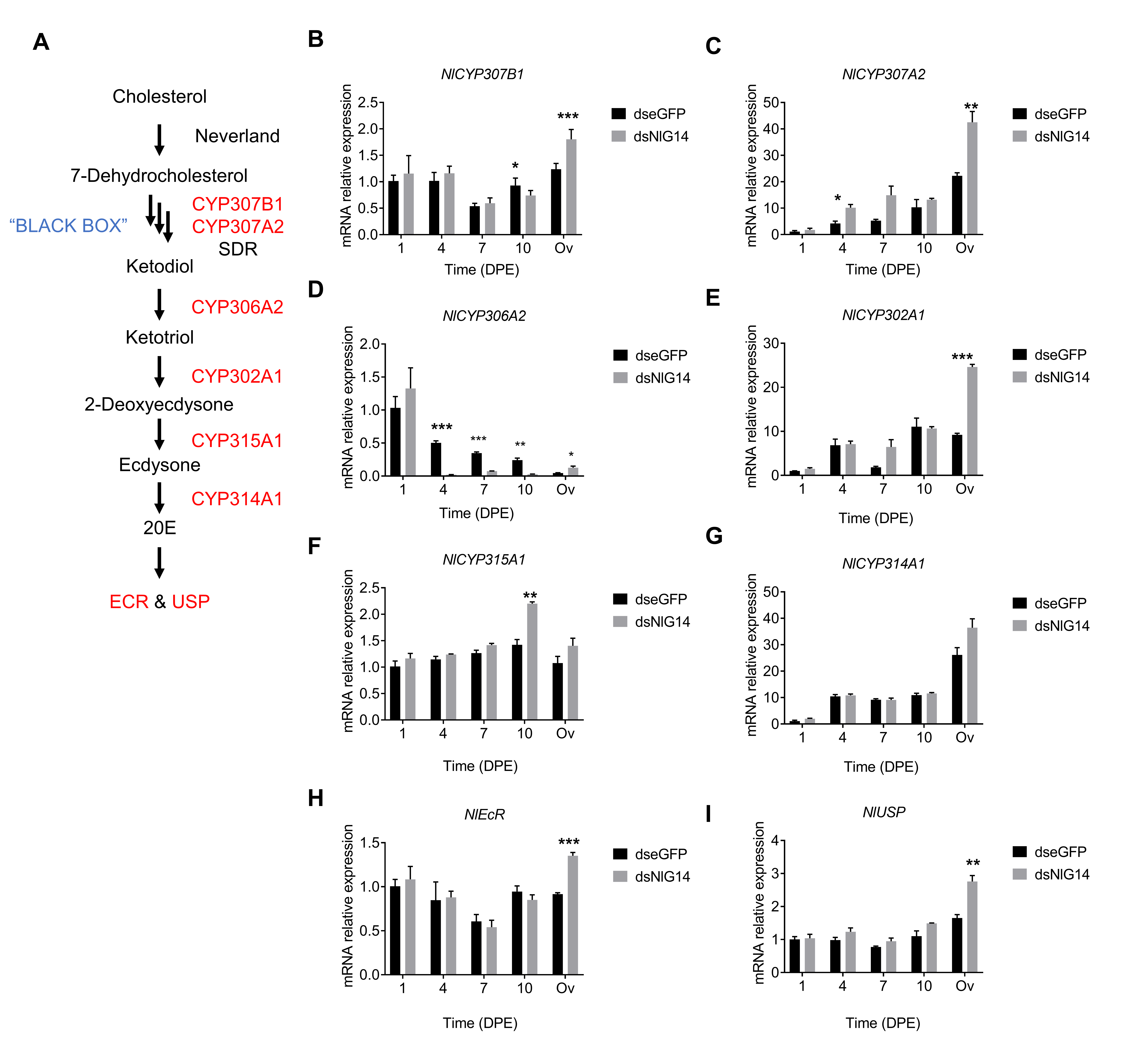

Supplement: S4 Fig — (A) Schematic sketch of ecdysteroid biosynthetic pathway in BPH. (B-I) The genes expression variations of whole body on 1, 4, 7, 10 DPE and ovary on 7 DPE. Data are mean±SE (n = 3). Ov, ovary. Significant differences were determined using Student’ s t-test: *P<0.05; **P<0.01; ***P<0.001. (TIF) [file pgen.1010704.s004.tif]

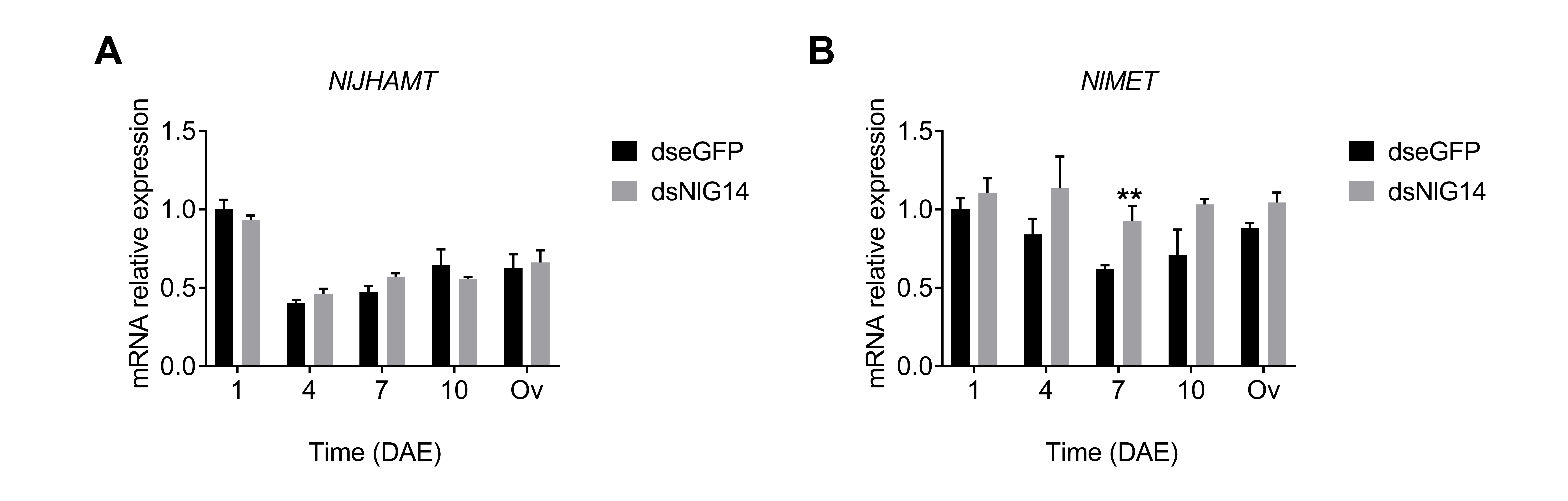

Supplement: S5 Fig — (A-B) The juvenile hormone biosynthetic (A) and receptor (B) genes expression variations of whole body on 1, 4, 7, 10 DPE and ovary on 7 DPE. Data are mean±SE (n = 3). Ov, ovary. Significant differences were determined using Student’ s t-test: **P<0.01. (TIF) [file pgen.1010704.s005.tif]
